# Comprehensive analysis of ceRNA network of ERCC4 in colorectal cancer

**DOI:** 10.7717/peerj.12647

**Published:** 2021-12-14

**Authors:** Huixin Hu, Songyi Liu, Aining Chu, Jing Chen, Chengzhong Xing, Jingjing Jing

**Affiliations:** 1 Tumor Etiology and Screening Department of Cancer Institute and General Surgery, the First Hospital of China Medical University, Shenyang, China; 2 Key Laboratory of Cancer Etiology and Prevention in Liaoning Education Department, the First Hospital of China Medical University, Shenyang, China; 3 Key Laboratory of GI Cancer Etiology and Prevention in Liaoning Province, the First Hospital of China Medical University, Shenyang, China; 4 Department of Anorectal Surgery in Liaoning Province, the First Hospital of China Medical of China Medical University, Shenyang, China

**Keywords:** Colorectal cancer, ceRNA, ERCC4, MALAT1, miR-200c-3p

## Abstract

**Objective:**

ERCC4 is one of the most significant molecules of Nucleotide Excision Repair (NER), which has been researched due to its high expression in colorectal cancer (CRC). This study aimed to find out the ceRNA (competitive endogenous RNA) network of *ERCC4* in CRC.

**Methods and Materials:**

Pan cancer mRNA expression of *ERCC4* was evaluated using TCGA database. The protein expression of ERCC4 was evaluated based on the Human Protein Atlas (HPA). We screened DElncRNAs and DEmiRNAs in two groups of *ERCC4*^high^ and *ERCC4*^low^ expression in CRC. Then a lncRNA-miRNA-*ERCC4* regulatory network was constructed based on DElncRNAs and DEmiRNAs using Starbase database and visualized by Cytoscape software. Kaplan-Meier analysis was performed to evaluate the prognostic value of the ceRNA network. Further, RT-PCR was performed to validate the expression of the representative molecules in the ceRNA network in CRC and normal tissues. The relationship between drug sensitivity and these molecules were also evaluated using RNAactDrug database.

**Results:**

*ERCC4* was overexpressed in a variety of tumors at mRNA levels, including CRC. High expression of ERCC4 was also observed on protein level in CRC. A total of 1,885 DElncRNAs and 68 DEmiRNAs were identified from CRC samples in *ERCC4*^high^ and *ERCC4*^low^ expression groups. Predicted by the Starbase database, we got interacting miRNAs and lncRNAs of *ERCC4* from the DEmiRNAs and DElncRNAs, and a lncRNA-miRNA-*ERCC4* regulatory network was constructed. Kaplan-Meier survival curves results showed that miR-200c-3p (hazard ratio [HR] = 0.62, *P* = 0.032), MALAT1 (HR = 1.54, *P* = 0.016), and AC005520.2 (hazard ratio [HR] = 1.75, *P* = 0.002) were significantly associated with the prognosis of CRC. After validation by RT-PCR, we found that *ERCC4* and MALAT1 were up-regulated in CRC compared with normal tissues, while miR-200c-3p was down-regulated. A strong negative correlation was observed between MALAT1 and miR-200c-3p. Drug sensitivity analysis showed that *ERCC4*, miR-200c and MALAT1 were all associated with Cisplatin.

**Conclusion:**

We constructed a ceRNA network of *ERCC4* in CRC, of which the MALAT1-miR-200c-3p-*ERCC4* axis may be involved in the development, prognosis and chemotherapy sensitivity of CRC. These findings might provide novel clues and insights on the molecular mechanisms of ERCC4 and NER pathway in CRC.

## Introduction

DNA damage caused by endogenous or exogenous genetic toxicants which contribute to genomic instability may lead to a variety of cancers (Iyama & Wilson, 2013). DNA repair systems play a critical role in maintaining the integrity and stability of the genome, of which nucleotide excision repair (NER) can identify and amend multiple types of potential DNA damage. The NER process consists of four main steps namely damage identification, damage partitioning and unwinding, damage incision and new strand synthesis (de Laat, Jaspers & Hoeijmakers, 1999; Liu et al., 2014; Slyskova et al., 2012). Different key proteins are involved in different NER steps. ERCC4 (XPF) is one of the indispensable molecules of NER, which can determine the activity of NER and is responsible for the removal of UV-C photoproducts and large volume adducts from DNA (Enzlin & Scharer, 2002). Besides, the heterodimer of ERCC4-ERCC1 is involved with the 5′ incision step of the NER pathway (Enzlin & Scharer, 2002). ERCC4-ERCC1 can repair damaged DNA in both replicating and non-replicating cells as a structure-specific endonuclease (Evans et al., 1997; Mu, Hsu & Sancar, 1996; Sijbers et al., 1996). Therefore, the abnormal expression of *ERCC4* could seriously affect the NER pathway.

Many researches have mentioned the role of *ERCC4* in cancers, such as bladder cancer, gastric cancer and oral cancer (Li & Ma, 2018; Qiu et al., 2014; Sa et al., 2020; Wang et al., 2010; Wei et al., 2014). Colorectal cancer (CRC) is a malignant tumor which is the third cause of cancer death in China (Zhang et al., 2021). Previous studies also focused on the relationship between *ERCC4* polymorphisms and the risk of CRC (He et al., 2014; Kabzinski et al., 2015; Yang, Li & Li, 2015). In recent studies, the post-transcriptional regulation of non-coding RNA plays an important role in the development of cancer (Anastasiadou, Jacob & Slack, 2018; Wei et al., 2017). Salmena et al. (2011) put forward the hypothesis of ceRNA, which expands our insight into the pathogenesis of CRC (Wei et al., 2017; Yuan et al., 2019). For example, Zhu et al. (2019) suggested that LINC00365 is an oncogene in CRC, which can regulate the expression of several mRNAs by sponging miRNAs. H19 was found to regulate PI3K-Akt signaling pathway through ceRNA network, and predict the poor prognosis of CRC (Zhong et al., 2019). Since *ERCC4* plays a significant role in CRC tumorigenesis, it is necessary to explore the related ceRNA regulatory network to better understand its mechanism in the development and progression of CRC. Although there have been studies investigating the relationship between *ERCC4* expression and the risk of CRC based on small sample size (Zhang et al., 2019), the ceRNA network regulation of *ERCC4* in CRC has not been identified.

In this study, we screened DElncRNAs and DEmiRNAs in two groups of *ERCC4*^high^ and *ERCC4*^low^ expression in CRC. Then a lncRNA-miRNA-*ERCC4* regulatory network was constructed based on DElncRNAs and DEmiRNAs using bioinformatics methods. Further, RT-PCR was performed to validate the expression of the representative molecules in the ceRNA network in CRC and normal tissues. The relationship between drug sensitivity and these molecules were also evaluated. Our research might be useful for further studies of the molecular mechanism of *ERCC4* in CRC.

## Materials and Methods

### Data preparation and processing

We firstly collected the gene expression information of 33 different kinds of tumors in TCGA database (http://cancergenome.nih.gov/). Further, the mRNA sequencing (mRNA seq) data of 698 CRC samples and miRNA sequencing (miRNA seq) data of 630 CRC samples were also gained from TCGA database. All raw RNA sequencing (RNA seq) data (lncRNAs, miRNAs, and mRNAs) were normalized as fragments per kilobase of exon model per million mapped fragment reads. The clinical information (survival status and survival time) were downloaded from UCSC XENA (https://xenabrowser.net/). Human Protein Atlas (HPA) database (http://www.proteinatlas.org/) was used to verify the protein expression level of ERCC4 in CRC.

### Identification of DElncRNAs and DEmiRNAs

We further performed differential expression analysis to identify DElncRNAs and DEmiRNAs in *ERCC4*^high^ and *ERCC4*^low^ CRC samples, with the median of *ERCC4* expression level as the cutoff value for grouping. We determined the DElncRNA with a threshold of | logFC | > 0.1 and *P* < 0.05, and a threshold of | logFC | > 0.5 and *P* < 0.05 for DEmiRNA. Volcano plots of the DElncRNAs and DEmiRNAs were visualized using GraphPad Prism 8 software (version 8.3.0).

### Construction of ceRNA network

Based on the hypothesis that lncRNAs compete with miRNAs as natural sponges in the cytoplasm and indirectly regulate mRNA expression, the ceRNA network was constructed through the following steps: (1) StarBase 3.0 (https://starbase.sysu.edu.cn/) was used to predict the potential miRNAs that interact with *ERCC4*. (2) The overlapping miRNAs intersected with DEmiRNA were selected and visualized using Venn diagram package in R software. (3) LncRNAs that potentially interact with overlapping miRNA were predicted using StarBase. (4) The intersection of the predicted lncRNAs and DElncRNAs was calculated and visualized by the Venn diagram. (5) Based on the identified lncRNAs and miRNAs, we constructed and visualized a lncRNA-miRNA-*ERCC4* triple regulation network using Cytoscape software (https://www.cytoscape.org/) (Shannon et al., 2003).

### Survival analysis

We performed Kaplan-Meier analysis and log-rank test on DElncRNAs, DEmiRNAs, and *ERCC4* in the ceRNA network using survminer package in R software, thus to determine their relationships with OS of CRC patients in TCGA database. The log-rank *P* value < 0.05 was considered statistically significant.

### Real-time PCR validation

We enrolled a total of 67 cases of CRC and adjacent normal tissue for validation, which were collected under enterectomy at the First Hospital of China Medical University. The clinical parameters of them were listed in File S1.

Total RNA was extracted using RNAiso Plus (Takara, Kusatsu, Japan) and converted into cDNA using PrimeScript RT Kit (Takara, Kusatsu, Japan). Amplification was performed with SYBR Green (SYBR Premix Ex Taq II; Takara, San Jose, CA, USA). Each response of lncRNA was normalized to β-actin, and U6 for miRNA. The study was approved by the Human Ethics Committee of China Medical University. The IRB approval number is 2016-155-2.

### Drug sensitivity analysis based on multiomics data

RNAactDrug (http://bio-bigdata.hrbmu.edu.cn/RNAactDrug) is a comprehensive database of relationship between RNAs and drug sensitivity, which integrated three large-scale pharmacogenomic databases respectively called Cancer Cell Line Encyclopedia (CCLE), Drug Sensitivity in Cancer (GDSC) and CellMiner. We explored the association between drug sensitivity and *ERCC4*, miR-200c and MALAT1 at expression, mutation, CNV, and methylation level. Pearson correlation analysis was used, and statistical significance was set at *P* < 0.05.

### Statistical analysis

The statistical analysis was conducted through SPSS 20.0 software (IL, Chicago) and R software. The difference of RNA expression between CRC and non-tumor tissues was compared by t-tests, and the fold change value was used to evaluate the difference. We applied correlation analysis to find out the Pearson correlation coefficients (r) and corresponding *P* value. *P* < 0.05 was considered as statistically significant.

## Results

### The expression and prognostic value of *ERCC4* in CRC

**Figure 1:**
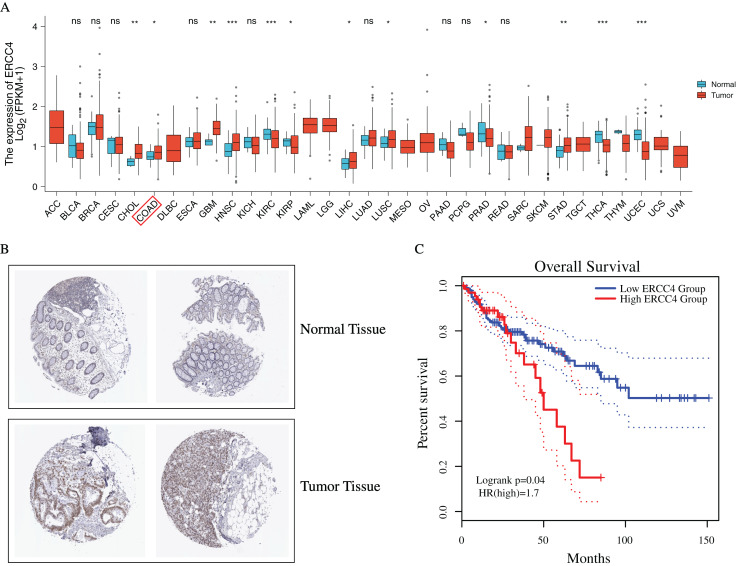
The expression and prognostic value of ERCC4 in CRC. (A) The mRNA expression level of ERCC4 in 33 different kinds of tumors based on TCGA database. (B) The protein expression level of ERCC4 in normal colorectal tissue and CRC tissue based on the Human Protein Atlas database. (C) The survival analysis of ERCC4 in colon cancer. *P* = 0.04. *: *P* < 0.05, **: *P* < 0.01, ***: *P* < 0.001.

To investigate the possible role of *ERCC4* in CRC, based on TCGA data, we found that *ERCC4* was overexpressed in many kinds of tumors on mRNA level, including CRC (Fig. 1A). In addition, based on the HPA database, high expression of ERCC4 was also observed on protein level in CRC (Fig. 1B). Since *ERCC4* was up-regulated in tumor specimens, we then investigated the clinical significance of *ERCC4* expression in CRC patients. According to the Kaplan-Meier survival curves, as shown in Fig. 1C, our results showed that higher expression of *ERCC4* was correlated with poor overall survival (OS) in colon cancer patients.

### Identification of DEmiRNAs and DElncRNAs

**Figure 2:**
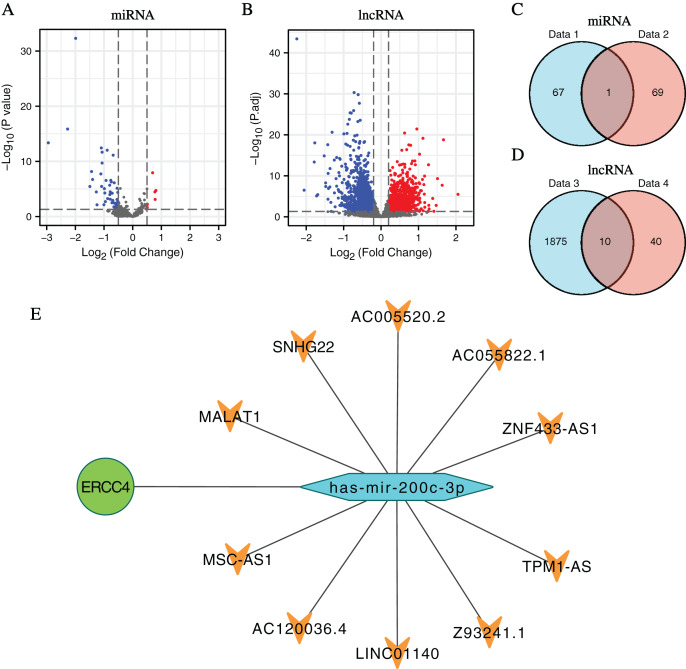
DElncRNAs and DEmiRNAs between the expression of ERCC4^high^ and ERCC4^low^ in CRC samples and the lnRNA-miRNA-ERCC4 triple regulatory network. (A) Visualized volcano plots of the DEmiRNAs in ERCC4^high^ and ERCC4^low^ CRC samples. (B) Visualized volcano plots of the DElncRNAs in ERCC4^high^ and ERCC4^low^ CRC samples. (C) The intersection of the predicted miRNA and DEmiRNA. (D) The intersection of the predicted lncRNA and DElncRNA. (E) The triple network of lncRNA-miRNA-ERCC4.

In total, 1,885 DElncRNAs (412 upregulated and 1,473 downregulated), 68 DEmiRNAs (7 upregulated and 61 downregulated) were identified from CRC samples in *ERCC4*^high^ and *ERCC4*^low^ expression groups (Figs. 2A, 2B, File S2).

### Construction of ceRNA network of *ERCC4*

Predicted by the Starbase database, we got interacting miRNAs of *ERCC4* from the DEmiRNAs, and only one overlapping miRNA (miR-200c-3p) was identified (Fig. 2C). Further, 10 lncRNAs interacting with miR-200c-3p were identified from DElncRNAs (Fig. 2D). Based on the theory of ceRNA regulatory network, and the obtained results of above DEmiRNAs and DElncRNAs, we constructed a lncRNA-miRNA-*ERCC4* regulatory network in Fig. 2E.

### Survival analysis of the molecules in the ceRNA network

**Figure 3:**
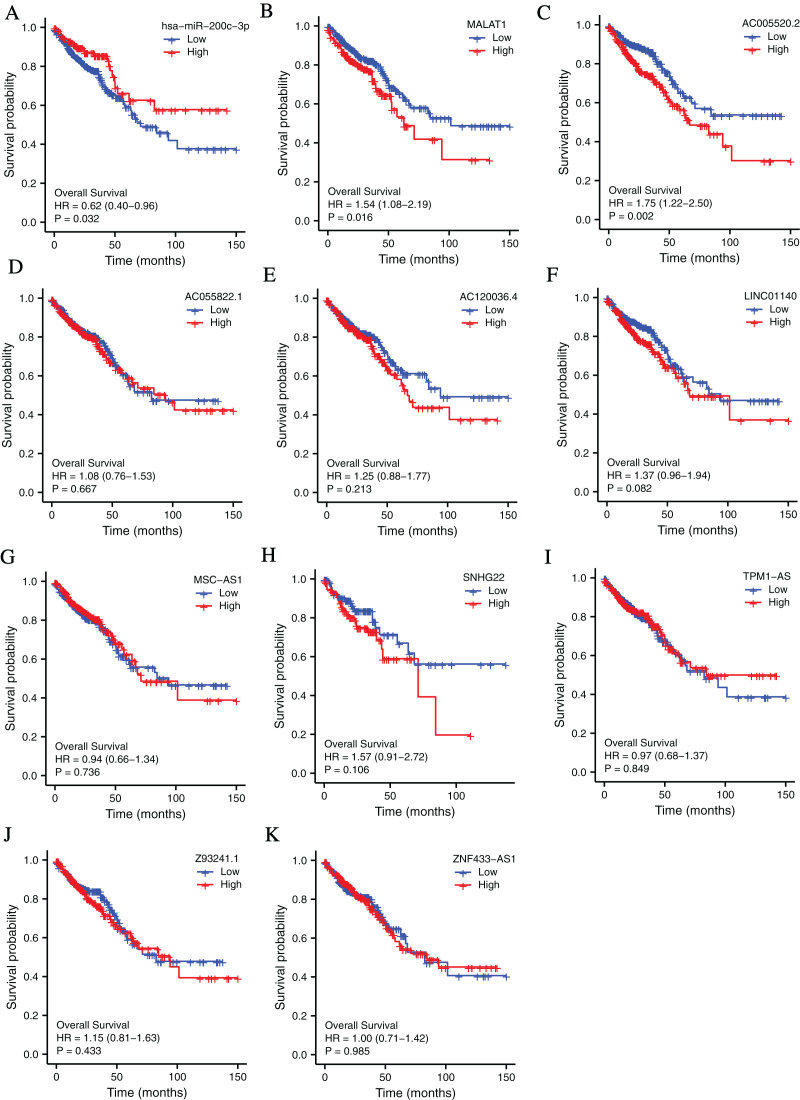
The lncRNAs and miRNAs in the ceRNA network and their relationships with CRC prognosis base on TCGA database. (A) Higher expression of has-miR-200c-3p had a longer OS in CRC. HR = 0.62, *P* = 0.032. (B) Lower expression of MALAT1 had a longer OS in CRC. HR = 1.54, *P* = 0.016. (C) Lower expression of AC005520.2 had a longer OS in CRC. HR = 1.75, *P* = 0.002. (D–K) AC055822.1, AC120036.4, LINC01140, MSC-AS1, SNHG22, TPM1-AS, Z9324.1 and ZNF433-AS1 are not related to CRC prognosis. *P* < 0.05 was statistically significant.

To investigate the clinical values of the molecules in the ceRNA network in CRC, Kaplan-Meier survival curves were used to evaluate the prognostic significance. The results showed that miR-200c-3p (hazard ratio [HR] = 0.62, *P* = 0.032), MALAT1 (hazard ratio [HR] = 1.54, *P* = 0.016), and AC005520.2 (hazard ratio [HR] = 1.75, *P* = 0.002) were significantly associated with the prognosis of CRC (Figs. 3A–3F).

### RT-PCR verification of the molecules in the ceRNA network

**Figure 4:**
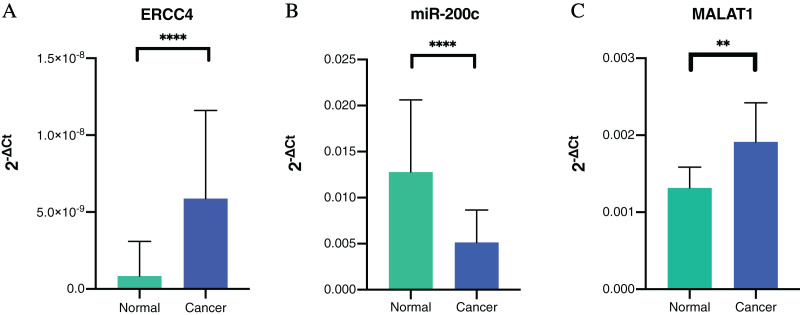
The expressions of ERCC4, miR-200c and MALAT1 in CRC and normal tissues based on RT-PCR. (A) ERCC4 was up-regulated in CRC compared with normal tissues. *P* < 0.05. (B) MiR-200c was down-regulated in CRC comparing with normal tissues. *P* < 0.05. (C) MALAT1 was up-regulated in CRC compared with normal tissues. *P* < 0.05.

To further verify our bioinformatic analysis results, we performed RT-PCR experiment to explore the expression of the molecules in the ceRNA network in CRC and normal tissues. Because MALAT1 showed a prognostic value in CRC, and it was a well-studied lncRNA in cancers, we selected *ERCC4*, miR-200c-3p and MALAT1 for verification. Through differential expression analysis, it could be found out that *ERCC4* and MALAT1 were up-regulated in CRC compared with normal tissues, while miR-200c-3p was down-regulated in CRC compared with normal tissues, which were all with statistical significance (Fig. 4).

**Figure 5:**
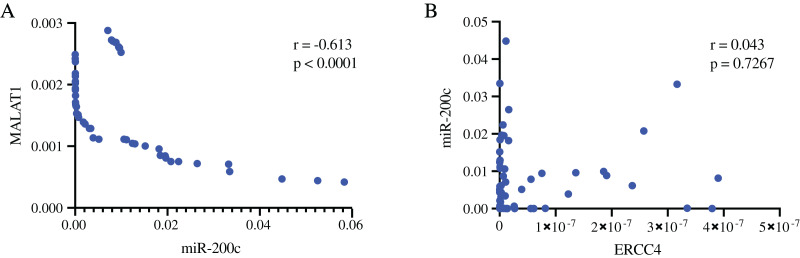
The associations of MALAT1, miR-200c-3p, and ERCC4 based on RT-PCR. (A) There is a strong negative correlation between MALAT1 and miR-200c-3p. r = −0.613, *P* < 0.0001. (B) The correlation between miR-200c-3p and ERCC4 was not statistically significant. *P* = 0.723.

In addition, we validated the association of MALAT1-miR-200c-3p-*ERCC4* by correlation analysis (Fig. 5). A strong negative correlation (r = −0.613) was found between MALAT1 and miR-200c-3p (*P* < 0.0001), while the correlation between miR-200c-3p and ERCC4 was not statistically significant (*P* = 0.723).

### The correlation between *ERCC4*, miR-200c-3p, MALAT1 and drug sensitivity

**Figure 6:**
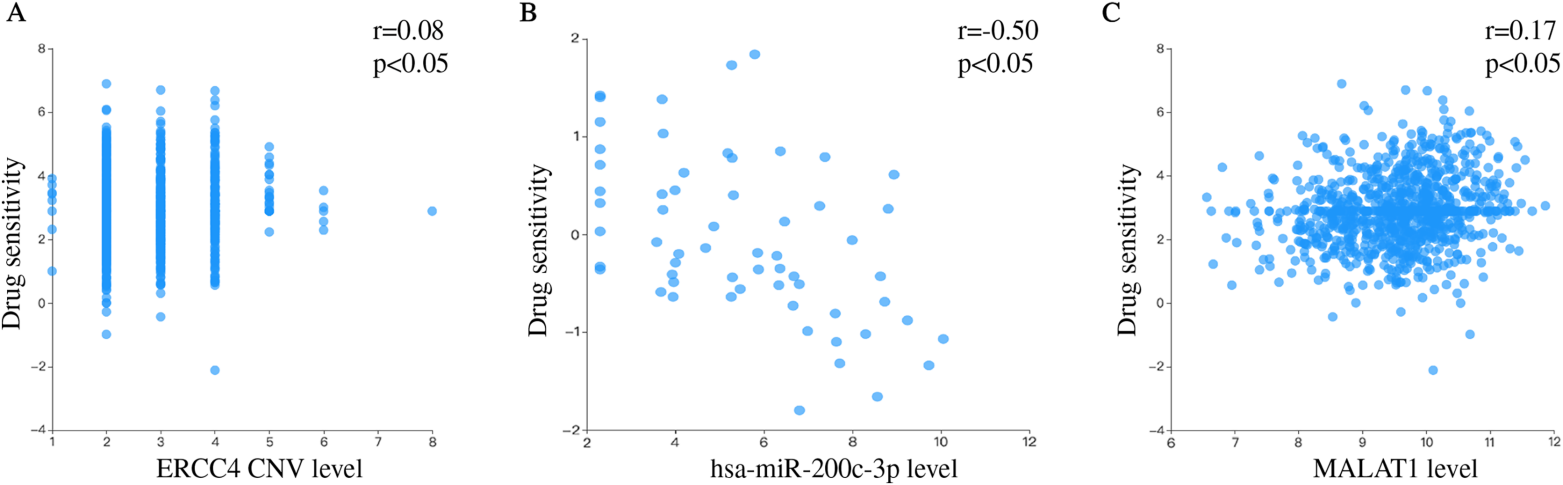
Drug sensitivity analysis of ERCC4, hsa-miR-200c-3p and MALAT1. (A) The correlation between drug sensitivity and ERCC4 (Copy number variations, CNV). Pearson r = 0.08. Pearson *P* < 0.05. (B) The correlation between drug sensitivity and hsa-miR-200c-3p (gene expression). Pearson r = −0.50. Pearson *P* < 0.05. (C) The correlation between drug sensitivity and MALAT1 (gene expression). Pearson r = 0.17. Pearson *P* < 0.05.

We separately explored the relationship between drug sensitivity and *ERCC4*, miR-200c and MALAT1 using RNAactDrug database. The results were shown in File S3. In addition, we found that *ERCC4*, miR-200c and MALAT1 were all associated with Cisplatin (Fig. 6).

## Discussion

NER pathway plays an important role in identifying and repairing DNA damage, in which ERCC4 is one of the unnegligible molecules. Abnormal expression of *ERCC4* can lead the imbalance between DNA damage and repair (Neven et al., 2018). *ERCC4* has been found with high-expression in cancer tissues, including CRC (McDaniel & Schultz, 2008). Although there have been a few studies on *ERCC4* in several cancers, its epigenetic regulation is still not fully understood. Therefore, we attempted to establish a *ERCC4*-related ceRNA triple network in CRC to provide a better understanding of the biological mechanisms of *ERCC4* in CRC.

Firstly, we evaluated the expression and prognostic value of *ERCC4* in CRC using TCGA data. *ERCC4* was found to be upregulated in CRC tissues compared with normal tissues both on mRNA and protein levels. It was also associated with prognosis of colon cancer patients. Then 1885 DElncRNAs and 68 DEmiRNAs were sorted out from CRC samples in *ERCC4*^high^ and *ERCC4*^low^ expression groups. One miRNA and 10 lncRNAs were then identified from the DElncRNAs and DEmiRNAs through bioinformatics prediction using Starbase database. Based on these results, a lncRNA-miRNA-*ERCC4* regulatory network was constructed. We also investigated the clinical values of the molecules in the ceRNA network in CRC, and found that miR-200c-3p, MALAT1 and AC005520.2 were significantly associated with the prognosis of CRC.

Further, we performed RT-PCR experiment to verify the expression of the molecules in the ceRNA network in CRC and normal tissues. By comprehensively considering the prognostic value and research status, we selected *ERCC4*, miR-200c-3p and MALAT1 for verification. Our results showed that *ERCC4* and MALAT1 were up-regulated in CRC, while miR-200c-3p was down-regulated in CRC. In addition, we validated the association of MALAT1-miR-200c-3p-*ERCC4* by correlation analysis. A strong negative correlation was observed between MALAT1 and miR-200c-3p. The drug sensitivity analysis showed that *ERCC4*, miR-200c and MALAT1 were all associated with Cisplatin. Therefore, the MALAT1-miR-200c-3p-*ERCC4* axis may be closely associated with the development, prognosis and chemotherapy sensitivity of CRC and deserve further study.

*ERCC4*, as a key gene in NER, is mainly involved in the removal of damage fragments (Friedberg, 2001). ERCC4 domain is also necessary to form a tight complex with ERCC1, a structure specific DNA repair endonuclease responsible for 5’-primer cleavages during DNA excision and repair (Tripsianes et al., 2005; Tsodikov et al., 2005). MALAT1 is located on human chromosome 11q13 and can generate an 8 kb length transcript of long non-coding and nuclear-retained (Ji et al., 2003; Lin et al., 2007; Zhang, Hamblin & Yin, 2017). It can act at the transcriptional, post-transcriptional and translational levels, thus it may be involved in various biological processes such as DNA damage repair, metabolism, and cell signaling (Castro-Oropeza et al., 2018). MiR-200c is highly studied because it has an impact on development, stemness, proliferation, epithelial–mesenchymal transition (EMT), metastasis and therapy resistance (Mutlu et al., 2016). MiR-200c is predicted to target *SIRT1* which was found to be negatively regulated by Poly polymerase-2, an enzyme that plays significant roles in the DNA damage response (Nabih, 2020). Therefore, these non-coding RNAs in ceRNA network may competitively regulate *ERCC4* to affect the balance between DNA damage and repair, and participate in the development of CRC.

MALAT1 as a highly conserved lncRNA, has been found to be overexpressed in several human neoplasms and to promote tumor cell invasion and metastasis (Castro-Oropeza et al., 2018). It has been reported regulating mRNA expression and then contributing to CRC progression by sponging miRNA in a few studies, like miR-26a/26b (Xu et al., 2020), miR-106b-5p (Zhuang et al., 2019), and miR-203 (Wu et al., 2018). The miR-200 family played an important role in EMT. It was mentioned that miR-200c-3p was not only differentially expressed in CRC, but also may inhibit CRC migration and invasion, and promote apoptosis after LPS stimulation (Huang et al., 2020; Jiang et al., 2020). The miR-200c was directly target *ZEB1* and *ZEB2* which are the *CDH1* transcription inhibitors. Inhibition of miR-200c reduces the expression of *CDH1* and therefore induces EMT (Gregory et al., 2008; Park et al., 2008). MALAT1 has been shown to induce EMT during endometriosis (Du et al., 2019) and metastasis in clear cell kidney carcinoma mouse models (Xiao et al., 2015) *via* the miR200/*ZEB2* axis by acting as a sponge for the miR200 family. Sreekumar R found that *ZEB2*-dependent EMT transcriptional programme drives therapy resistance by activating nucleotide excision repair genes *ERCC1* and *ERCC4* in CRC (Sreekumar et al., 2021). These results demonstrated that MALAT1, miR-200 and *ERCC4* were all connected to the EMT. Therefore, the MALAT1-miR-200c-3p-*ERCC4* axis may be involved in the malignant behaviour of CRC by affecting EMT.

DNA repair has an essential role in resistance to platinum agents. Platinum agents inhibit DNA replication and transcription by binding to DNA molecules in the form of platinum DNA adducts (Altaha et al., 2004). ERCC4 is an important protein in the NER pathway, which is a major part of the DNA repair pathway (Fuss & Cooper, 2006; Gillet & Scharer, 2006). CRC cells expressing *ZEB2* developed resistance to oxaliplatin while *ERCC4* is induced upon *ZEB2* expression (Sreekumar et al., 2021). Forced expression of miR-192-5p in SGC7901/DDP cells can significantly inhibit the expression of *ERCC4*, making the gastric cancer cells more sensitive to cisplatin *in vitro* and *in vivo* (Xie et al., 2019). MiR-138-5p regulated the cisplatin resistance by modulating the expression of the DNA repair proteins ERCC4 and ERCC1 in gastric cancer cells (Ning et al., 2019). During repeated treatments of oxaliplatin, the down-regulated expression of miR-200c could lead the resistance to oxaliplatin (Tanaka et al., 2015). It is worth noting that miR-200c and miR-200b can reverse the resistance of epithelial ovarian cancer to cisplatin by targeting DNA methyltransferase (DNMT) (Liu et al., 2019a). Zhang, Li & Zhang (2020) found that MALAT1 modulated *ZFP91* through sponging miR-22-3p to promote oxaliplatin resistance in gastric cancer cells. MALAT1 was significantly up-regulated in CRC cells and tissues which wre resistant to oxaliplatin. MALAT1 modulated *ADAM17 via* sponging miR-324-3p to enhance oxaliplatin resistance in CRC cells (Fan et al., 2020). Liu et al. (2019b) has fingered out that the down-regulated of MALAT1 improved the chemotherapeutic drug sensitivity and inhibited the cisplatin resistance of the bladder cancer. Interestingly, our results showed that *ERCC4*, miR-200c and MALAT1 were all associated with Cisplatin, which suggested that MALAT1-miR-200c-3p-*ERCC4* axis may play a role in the Platinum-based chemotherapy for CRC patients.

However, the limitation of this study should be acknowledged. The sample size is relatively small and a larger sample verification is still needed. The biological relationship of MALAT1-miR-200c-3p-*ERCC4* axis and the biological functions they participate in are still unclear, and further *in vivo* and *in vitro* studies are needed. It would make more sense if we could obtain the chemotherapy information of patients in TCGA. We will further explore the underlying association between platinum-based chemotherapy and the network in future research.

## Conclusion

In summary, we constructed a ceRNA network of *ERCC4* in CRC, of which the MALAT1-miR-200c-3p-*ERCC4* axis may be involved in the development, prognosis and chemotherapy sensitivity of CRC. This study will help to further reveal the molecular mechanism of *ERCC4* in CRC and provide new clues and insights for the further study of NER pathway. However, our results are only based on bioinformatic analysis and tissue validation of small samples, further *in vivo* and *in vitro* studies are needed to investigate the biological relationship of MALAT1-miR-200c-3p-*ERCC4* axis and its biological function.

## Supplemental Information

10.7717/peerj.12647/supp-1 Supplemental Information 1 The clinical parameters of ERCC4, miR-200c and MALAT1.

10.7717/peerj.12647/supp-2 Supplemental Information 2 The list of 68 DEmiRNAs and 1885 DElncRNAs.

10.7717/peerj.12647/supp-3 Supplemental Information 3 The correlations between ERCC4, miR-200c, MALAT1 and drug sensitivity.

## Abbreviations

ACC: Adrenocortical carcinoma
BLCA: Bladder Urothelial carcinoma
BRCA: Breast Invasive Carcinoma
ceRNA: competitive endogenous RNA
CCLE: Cancer Cell Line Encyclopedia
CESC: Cervical squamous cell carcinoma and endocervical adenocarcinoma
CHOL: Cholangiocarcinoma
COAD: Colon adenocarcinoma
CRC: Colorectal cancer
DLBC: Lymphoid Neoplasm Diffuse Large B-cell Lymphoma
DNMT: DNA methyltransferase
EMT: epithelial-mesenchymal transition
ESCA: Esophageal carcinoma
GBM: Glioblastoma multiforme
GDSC: Drug Sensitivity in Cancer and CellMiner
HNSC: Head and Neck squamous cell carcinoma
HPA: the Human Protein Atlas
KICH: Kidney Chromophobe
KIRC: Kidney renal clear cell carcinoma
KIRP: Kidney renal papillary cell carcinoma
LAML: Acute Myeloid Leukemia
LGG: Brain Lower Grade Glioma
LIHC: Liver hepatocellular carcinoma
LUAD: Lung adenocarcinoma
MESO: Mesothelioma
NER: Nucleotide Excision Repair
OS: Overall survival
OV: Ovarian serous cystadenocarcinoma
PAAD: Pancreatic adenocarcinoma
PCPG: Pheochromocytoma and Paraganglioma
PRAD: Prostate adenocarcinoma
READ: Rectum adenocarcinoma
SARC: Sarcoma
SKCM: Skin Cutaneous Melanoma
STAD: stomach and esophageal carcinoma
TGCT: Testicular Germ Cell Tumors
THCA: Thyroid carcinoma
THYM: Thymoma
UCEC: Uterine Corpus Endometrial Carcinoma
UCS: Uterine Carcinosarcoma
UVM: Uveal melanoma

## Additional Information and Declarations

### Data Availability

The following information was supplied regarding data availability: The basic information of the cases and original data of qRT-PCR are available in the Supplemental Files.
